# SpinachBase: a central portal for spinach genomics

**DOI:** 10.1093/database/baz072

**Published:** 2019-06-18

**Authors:** Keeley Collins, Kun Zhao, Chen Jiao, Chenxi Xu, Xiaofeng Cai, Xiaoli Wang, Chenhui Ge, Shaojun Dai, Quanxi Wang, Quanhua Wang, Zhangjun Fei, Yi Zheng

**Affiliations:** 1 Boyce Thompson Institute for Plant Research, Ithaca, NY 14853, USA; 2 Development and Collaborative Innovation Center of Plant Germplasm Resources, College of Life Sciences, Shanghai Normal University, Shanghai 200234, China; 3 USDA-Agricultural Research Service, Robert W. Holley Center for Agriculture and Health, Ithaca, NY 14853, USA

## Abstract

Spinach (*Spinacia oleracea* L.) is a nutritious vegetable enriched with many essential minerals and vitamins. A reference spinach genome has been recently released, and additional spinach genomic resources are being rapidly developed. Therefore, there is an urgent need of a central database to store, query, analyze and integrate various resources of spinach genomic data. To this end, we developed SpinachBase (http://spinachbase.org), which provides centralized public accesses to genomic data as well as analytical tools to assist research and breeding in spinach. The database currently stores the spinach reference genome sequence, and sequences and comprehensive functional annotations of protein-coding genes predicted from the genome. The database also contains gene expression profiles derived from RNA-Seq experiments as well as highly co-expressed genes and genetic variants called from transcriptome sequences of 120 cultivated and wild *Spinacia* accessions. Biochemical pathways have been predicted from spinach protein-coding genes and are available through a pathway database (SpinachCyc) within SpinachBase. SpinachBase provides a suite of analysis and visualization tools including a genome browser, sequence similarity searches with BLAST, functional enrichment and functional classification analyses and functions to query and retrieve gene sequences and annotations.

## Introduction

Spinach (*Spinacia oleracea* L.) is an economically and nutritionally important vegetable crop belonging to the Amaranthaceae family of Caryophyllales, the basal order of core eudicots (1, 2). The annual worldwide gross production of spinach in 2016 was about 26 million tonnes, of which around 92% was produced in China (FAOSTAT; http://faostat3.fao.org). Spinach is a green, leafy vegetable commonly used in salads, cooked dishes, etc., and is an important source of vitamins and minerals such as carotenoids, vitamin C, vitamin K, folic acid, iron and calcium (USDA Nutrient Database; http://ndb.nal.usda.gov/ndb/search/list). More than 2000 spinach germplasm accessions have been collected throughout the world and are stored in gene banks, and their passport data are available in the International Spinach Database (https://ecpgr.cgn.wur.nl/LVintro/spinach/).

Spinach is an annual or biennial diploid species (2n = 2× = 12) with an estimated genome size of 989 Mb (3). Thanks to the rapid advances in sequencing technologies, recently genomic resources of spinach have been developed and become publicly available. In an effort to generate evidence supporting the separation of Caryophyllales before the split of asterids and rosids, Dohm *et al*. (1) developed a preliminary assembly for spinach cultivar Viroflay that covered about half of the spinach genome. Genome sequence of Viroflay and the gene set predicted from the genome (4) are available at the *Beta vulgaris* Resource (http://bvseq.boku.ac.at). Recently, a draft genome of a sibling inbred spinach line, ‘Sp75’, has been developed using the whole genome shotgun approach combined with BioNano Genomics optical maps and a high-density genetic map (2). This high-quality draft genome has served as a robust reference for spinach research and breeding, as well as plant comparative genomic and evolutionary studies. In addition, transcriptomes of 120 cultivated and wild *Spinacia* accessions were sequenced and comparison of these transcriptome sequences has resulted in a large number of variants in the transcribed regions of the spinach genome, as well as a large dataset of gene expression profiles (2, 5). Comprehensive analyses of these transcriptome variants have facilitated our understanding of spinach genetic diversity and domestication, and identified potential genome regions that have been affected by human selection, as well as those that are tightly associated with important agronomical traits such as bolting, flowering and leaf numbers (2).

Both the ‘Sp75’ reference genome and the transcriptome sequences of cultivated and wild spinach accessions have proved to be valuable resources for biological discovery and germplasm improvement in spinach. Furthermore, additional genome resources of spinach are expected to be developed in the near future, thanks to the recent advances of third-generation sequencing technologies. To efficiently disseminate and integrate these large-scale heterogeneous datasets, we have built the SpinachBase (http://spinachbase.org), a central portal for spinach genomics. SpinachBase has been implemented using Tripal, a widely used toolkit for constructing web-based genomic and genetic databases (6, 7). Tripal acts as an interface between Drupal, a popular website creation and content management system (http://drupal.org), and the Chado database schema. Chado is a generic, modular, ontology-driven and open-source database schema, and provides tables and definitions that describe how and what data are to be kept and retrieved from a database management system (8). Tripal is currently the basis for implementing many plant genome databases such as the Genome Database for Rosaceae (9) and Cucurbit Genomics Database (CuGenDB) (10). Tripal also contains dozens of extension modules that can integrate various analysis results, and these extension modules can be easily adopted in other genome databases built with Tripal. In SpinachBase, we implemented several extension modules to integrate the comprehensive functional annotation information and RNA-Seq expression profiles, as well as functional enrichment analysis tools. We also developed the ‘co-expression’ module to manage co-expressed genes identified based on the RNA-Seq expression profiles in the 120 cultivated and wild *Spinacia* accessions.

### Database description

#### Genome sequence and annotation

A total of 25 495 protein-coding genes were predicted from the draft genome of spinach ‘Sp75’ (2). The spinach genome sequences, coding sequences (CDS) and protein sequences of predicted spinach genes, a genome feature file in gff3 format (generic feature format), and functional descriptions of predicted genes can be downloaded from the ‘download’ page of SpinachBase. The sequences of chromosomes, scaffolds, CDS and proteins were imported into the Chado database using the Tripal ‘Data Loader’ module.

A standard and unified pipeline described in our previous study (10) was used to comprehensively annotate spinach protein-coding genes. Briefly, protein sequences of spinach genes were compared against the GenBank nr, UniProt (TrEMBL/SwissProt) and Arabidopsis protein databases using the BLAST program (11). Functional domains were then identified from spinach proteins by comparing their sequences to the InterPro database using InterProScan (12). Gene ontology (GO) terms were assigned to spinach protein-coding genes using the Blast2GO program (13) based on the comparison results against the nr and the InterPro domain databases. A set of concise and informative functional descriptions were assigned to spinach genes using the AHRD program (https://github.com/groupschoof/AHRD) based on the BLAST results against the UniProt (TrEMBL/Swiss-Prot) and Arabidopsis protein databases. With the assistance of the Tripal ‘Analysis’ extension modules, the homologs derived from top BLAST hits, GO terms and InterPro domains assigned to the protein-coding genes were deposited into the SpinachBase and displayed on the gene feature page.

**Figure 1:**
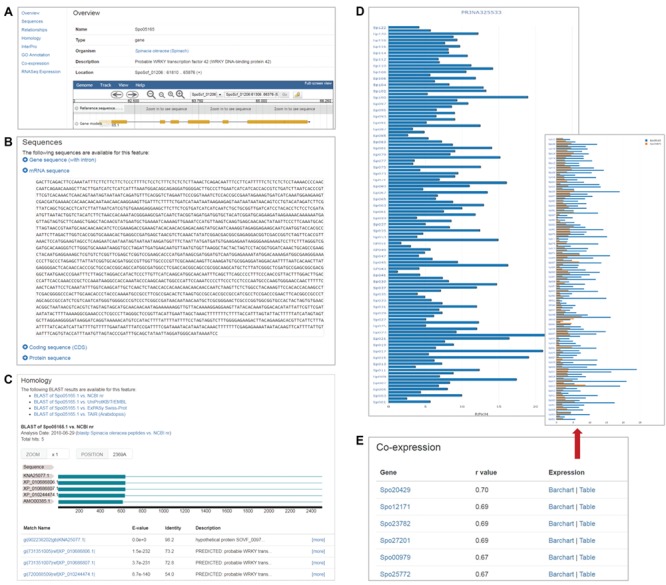
Gene feature page in SpinachBase. **(A)** Overview of a gene feature page. The page contains basic information about the gene feature including gene name, organism, gene location on the genome and functional description. The gene structure can be seen in JBrowse embedded in the page. The left panel provides links to different sections with different content types. **(B)** Section on the gene feature page showing sequence information of the feature. **(C)** Section on the gene feature page listing the homologs of the gene identified by BLAST. **(D)** Section on the gene feature page listing the normalized expression values from a selected project. **(E)** Section on the gene feature page showing genes that are co-expressed with a specific gene.

The gene feature page displays various information about a specific spinach gene in different sections. Users can access the information through the links on the left panel (Figure 1). The overview section of the feature page displays basic gene feature information and provides a view of the feature in a genome browser, which is implemented using Jbrowse (14) and embedded in the gene feature page (Figure 1A). The remaining sections include those that display sequence information (Figure 1B), annotated GO terms associated with the gene feature, homology information in related or model species (Figure 1C), associated InterPro domains, relationships to other features, RNA-Seq expression profiles (Figure 1D) and co-expressed genes (Figure 1E).

#### RNA-Seq expression

Strand-specific RNA-Seq data of 120 cultivated and wild *Spinacia* accessions reported in Xu *et al*. (2) were downloaded from National Center for Biotechnology Information (NCBI) Sequence Read Archive (SRA) and processed using a unified pipeline described previously (10) to derive gene expression profiles. Briefly, adapter sequences and low quality bases were removed from the raw RNA-Seq reads using Trimmomatic v0.32 (15), and the trimmed reads shorter than 80 bp were discarded. The remaining high quality reads were aligned to the SILVA rRNA database (16) using Bowtie v1.1.2 (17) allowing up to three mismatches, and the mapped reads were removed. The final cleaned reads were aligned to the spinach reference genome using HISAT (18) allowing up to two mismatches. The number of reads (read count) mapped to each predicted spinach gene model was derived, and then normalized to FPKM (fragments per kilobase of exon per million mapped fragments). To display the RNA-Seq expression profiles, we implemented the ‘SRA’ and ‘RNA-Seq’ modules developed by our group (10) in SpinachBase. The project, sample and experiment information can be managed in SpinachBase using the ‘SRA’ module, and the raw counts, FPKM expression values were loaded into and displayed in SpinachBase using the ‘RNA-Seq’ module. Users can access the RNA-Seq expression through the gene feature page. To display the expression profiles in the 120 different *Spinacia* accessions, the ‘RNA-Seq’ module was modified so the expression values are displayed in a vertical bar chart (Figure 1D). The normalized expression value in a specific accession is displayed on mouse-over of the corresponding bar in the chart.

#### Co-expressed genes

Spearman correlation coefficient (*r*) was calculated for each pair of genes based on their expression profiles (FPKM) in the 120 *Spinacia* accessions. Only gene pairs with *r* values greater than 0.65 were kept as co-expressed genes. To manage these co-expressed genes, we developed a ‘co-expression’ module, which was used to import co-expressed genes into the Chado database. Genes that are co-expressed with a specific query gene can be accessed under the ‘co-expression’ section of the feature page of the said gene. The list of genes co-expressed with the query gene is provided in a table and ordered by *r* values. Users can access expression profiles (bar chart or table) of co-expressed gene pairs through links provided in the table (Figure 1E).

#### Genetic variants

**Figure 2:**
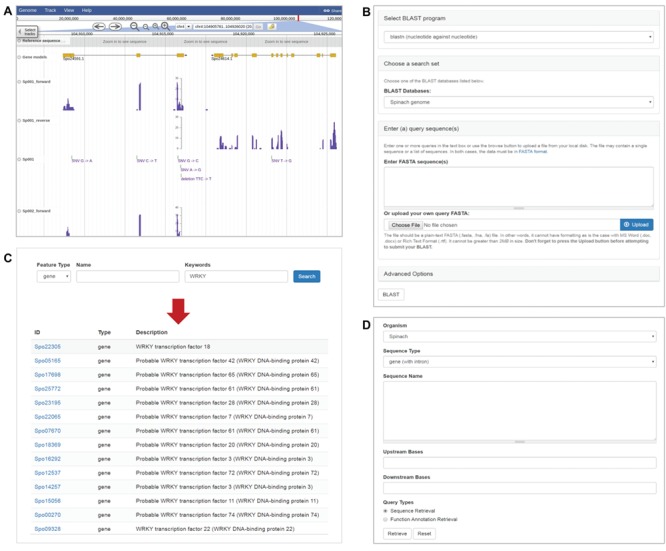
Tools in SpinachBase. **(A)** Display of strand-specific RNA-Seq expression profiles and genetic variants in JBrowse. **(B)** Query interface of the BLAST tool. **(C)** Query interface and result page of the feature search function. **(D)** Interface of the batch query.

Transcriptome sequences of the 120 *Spinacia* accessions were also used to call genetic variants, mainly single nucleotide polymorphisms and small indels, using GATK (19) following the online best practices protocol with recommended parameters for RNA-Seq data (https://software.broadinstitute.org/gatk/best-practices/). Briefly, the cleaned RNA-Seq reads were aligned to the spinach reference genome using STAR with the 2-pass method (20). The alignment files in SAM format (21) were further processed to add read groups, mark duplicates and create index using Picard (http://broadinstitute.github.io/picard). GATK (19) was then used to perform read splitting, base recalibration and variant calling based on the sorted alignment file. A total of 751 189 variants were identified among the 120 *Spinacia* accessions. These variants were integrated into SpinachBase and displayed on the genome browser (Figure 2A).

#### Metabolic pathways

Spinach enzymes and metabolic pathways were predicted from the protein-coding genes using Pathway Tools (22). A file in the PathoLogic format (22) was prepared, which included gene functional descriptions assigned by AHRD, GO terms assigned by Blast2GO and enzyme commission numbers derived from the top hits in the UniProt (TrEMBL/SwissProt) database. The file was processed by Pathway Tools, which predicted a total of 357 metabolic pathways in spinach. A pathway database, SpinachCyc, was built based on these predicted pathways using the web server of Pathway Tools (22). The SpinachCyc database is linked through the ‘Pathway’ tab in the main menu of the SpinachBase.

### Tools in SpinachBase

SpinachBase contains several data visualization, analysis and query tools located under the ‘Tools’ and ‘Genome’ tabs in the main menu. These tools include a genome browser, sequence similarity searches with BLAST, a gene feature search function, a batch query function, GO term and pathway enrichment analyses and gene functional classification.

#### Genome browser

The genome browser in SpinachBase was implemented using JBrowse (14), which allows users to visualize features of the spinach reference genome. Each chromosome or scaffold can be selected from a drop-down menu, and the browser displays information about the sequence and corresponding gene models. JBrowse in SpinachBase can be accessed under the ‘Genome’ tab of the main menu. In addition, as mentioned above, the gene feature page also has JBrowse embedded in the overview section to display the gene location and structure. The gene expression profiles and genetic variants are also displayed in JBrowse. To display the strand-specific RNA-Seq expression profiles in JBrowse, the read alignment file in BAM format (21) for each accession was split into two BAM files containing reads aligned to the spinach genome in forward and reverse directions, respectively. The resulting BAM files were converted to coverage tracks in bigWig format using deepTools2 (23), and these coverage tracks were then loaded onto JBrowse. To display genetic variants, the variants called by GATK in a VCF format file were indexed using VCFtools (24) and loaded onto JBrowse (Figure 2A). We also implemented the faceted selector based on the metadata of the 120 accessions, such as their countries of origins and geographic regions, to facilitate the selection of accessions for displaying expression and variant tracks.

#### Sequence similarity searches

SpinachBase allows similarity searches against spinach genome, mRNA and protein sequences using BLAST, which is one of the most frequently used tools in genome databases. To provide the BLAST search function, a modified version of the Tripal ‘BLAST UI’ module was implemented in SpinachBase. The ‘BLAST UI’ allows users to paste the query sequences or upload a file containing the sequences for BLAST against the spinach sequences. The query form of the ‘BLAST UI’ module was modified to integrate options of BLAST programs and different types of spinach sequences into one single form (Figure 2B). The BLAST search outputs tabular results available for download in different formats (HTML, XML, GFF3 and TSV). The BLAST result page includes a table listing all the hits, with each row of the table showing one blast hit information. The alignment between the query and hit sequences is displayed when clicking the particular row. In addition, an image is provided to show the coordinate relationship between the query and the hit, as well as color-ranked bit score for the BLAST hit, which helps to evaluate the quality of the BLAST results.

**Figure 3:**
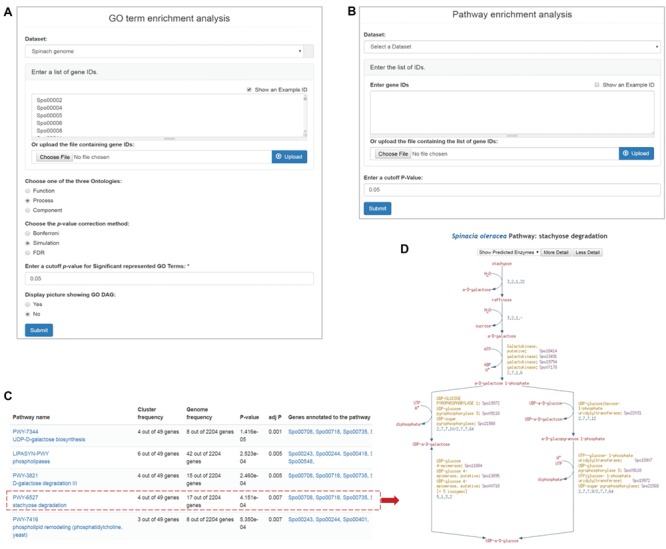
Enrichment analysis tools in SpinachBase. Query interfaces of ‘GO term enrichment analysis’ **(A)** and ‘Pathway enrichment analysis’ **(B)** tools. **(C)** Result page of ‘Pathway enrichment analysis’. Pathway names are linked to the specific pathway pages in the SpinachCyc database **(D)**.

#### Gene and function searches

The gene/function search tool is the main entrance to the gene feature page in SpinachBase. The tool was implemented using the Apache Solr search engine (http://lucene.apache.org/solr). With this powerful search engine, the search index was built based on the full text of the comprehensive functional annotations, including functional descriptions assigned by AHRD, homologous genes identified by BLAST, GO terms and InterPro domains. The search tool allows for an efficient retrieval of the spinach genome features. The features can be searched using filters such as feature type, name and keyword. Keyword can be any word or word combination of aforementioned functional annotations. The search tool outputs a table with names, types and functional descriptions of the matched features, which are linked back to the corresponding gene feature pages (Figure 2C).

In addition to the gene/function search tool, SpinachBase also has a batch query function built with the Tripal “Sequence Retrieval” module (7). When supplying a list of genes, the batch query function allows for retrieving sequences or functional annotations of these genes (Figure 2D).

#### Functional enrichment analyses

Functional enrichment analyses are frequently used in genomic and functional genomic studies, which help researchers to infer biologically meaningful insights for a list of interesting genes. In SpinachBase, we implemented the ‘GO enrichment analysis’ and the ‘pathway enrichment analysis’ tools using the Tripal ‘GO tool’ and ‘Pathway tool’ extension modules that were originally developed in CuGenDB (10). The ‘GO enrichment analysis’ tool allows users to supply a list of genes, select one of the three GO categories (biological process, molecular function or cellular component) and upon submission obtain a list of GO terms significantly enriched in the submitted genes compared to all genes predicted in the spinach genome (Figure 3A). The ‘pathway enrichment analysis’ tool allows users to identified significantly enriched metabolic pathways from a list of genes (Figure 3B). The output page of this tool includes a table listing the enriched pathways, the associated adjusted enrichment p-values, and gene IDs associated with the pathways (Figure 3C), with gene IDs linked to their feature pages and pathway IDs linked to the pathway page in the SpinachCyc database (Figure 3D).

#### Gene functional classification

In addition to the ‘GO enrichment analysis’ tool, the ‘GO tool’ extension module also provides a tool that can classify a list of interesting genes into different functional categories. The classification is based on a set of plant-specific GO subsets (http://www.geneontology.org/page/go-subset-guide). The ‘gene functional classification’ tool outputs a table that lists the GO subsets and their description and categories (biological process, molecular function or cellular component), number of genes assigned to each GO subset and the list of the assigned gene IDs.

## Conclusion

We have developed the SpinachBase (http://spinachbase.org), which serves as a central portal for spinach genomic data. Currently, the SpinachBase integrates sequences of spinach genome and protein-coding genes, comprehensive functional annotations and biochemical pathways predicted from the protein-coding genes, and analytical tools to assist research and breeding in spinach. We have also implemented a modified version of the ‘RNA-Seq’ module (10) to manage gene expression profiles, and a genome browser to display genetic variants called from transcriptome sequences of 120 cultivated and wild *Spinacia* accessions. It is worth noting that a new ‘co-expression’ module has been developed in SpinachBase, which can manage co-expressed genes inferred from the RNA-Seq expression profiles. This ‘co-expression’ module has been packed as a Tripal extension, and thus can be implemented in other genomic databases developed using the Tripal system. SpinachBase fills an important role in facilitating spinach research by enabling the efficient usage of genomic data for spinach breeding, comparative studies and further genomic studies. This new database is a central, easy to access repository with user-friendly interfaces and many tools for query and analyses of spinach genomic data. The use of Tripal to build the database also allows for any future demands of potentially storing new data types and developing new interfaces or tools to meet the demands of the spinach community.

## Notes

**Database URL**:http://spinachbase.org
